# Dielectric Properties of Glass Beads with Talc as a Reference Material for Calibration and Verification of Dielectric Methods and Devices for Measuring Soil Moisture

**DOI:** 10.3390/ma13081968

**Published:** 2020-04-23

**Authors:** Justyna Szerement, Hironobu Saito, Kahori Furuhata, Shin Yagihara, Agnieszka Szypłowska, Arkadiusz Lewandowski, Marcin Kafarski, Andrzej Wilczek, Jacek Majcher, Aleksandra Woszczyk, Wojciech Skierucha

**Affiliations:** 1 Institute of Agrophysics, Polish Academy of Sciences, Doświadczalna 4, 20-290 Lublin, Poland; a.szyplowska@ipan.lublin.pl (A.S.); a.lewandowski@elka.pw.edu.pl (A.L.); m.kafarski@ipan.lublin.pl (M.K.); a.wilczek@ipan.lublin.pl (A.W.); a.woszczyk@ipan.lublin.pl (A.W.); w.skierucha@ipan.lublin.pl (W.S.); 2 Department of Physics, Tokai University, 4-1-1 Kitakaname, Hiratsuka-shi, Kanagawa 259-1292, Japan; hilonobusaito@gmail.com (H.S.); frht.khr@gmail.com (K.F.); yagihara@keyaki.cc.u-tokai.ac.jp (S.Y.); 3 Institute of Electronic Systems, Warsaw University of Technology, Nowowiejska 15/19, 00-665 Warsaw, Poland; 4 Department of Electrical Engineering and Electrotechnologies, Lublin University of Technology, Nadbystrzycka 38A, 20-618 Lublin, Poland; j.majcher@pollub.pl

**Keywords:** seven-rod probe, dielectric properties, frequency-domain reflectometry, dielectric permittivity, moisture, salinity

## Abstract

This paper presents dielectric measurements of talc, glass beads, and their mixtures under different moisture and salinity levels. The measurements were conducted using a prototype seven-rod probe (15 mm long central rod) connected to a single port of vector network analyzer. The samples were moistened with distilled water and KCl solutions in order to obtain six different moisture content levels. The complex dielectric permittivity was determined from vector network analyzer reflection-coefficient measurements based on the open-water-liquid calibration procedure. Next, the fitting of volumetric water content–real part of dielectric permittivity calibration curves was performed for each material at selected frequencies, and the obtained relations were compared with well-known calibration equations. Additionally, a salinity index for the tested materials was calculated. It was concluded that pure talc is not an optimal material for the calibration and verification of dielectric methods. The calibration curves obtained for glass beads and the mixtures of glass beads with talc gave results close to well-known reference calibration functions. Additionally, the addition of talc caused the data points to be less scattered. Moreover, the values of the salinity index for the tested materials were in a good agreement with literature data for sand. The obtained results indicated that glass beads with the addition of talc can be used as a reference material for the calibration and verification of dielectric methods and devices for soil moisture measurement.

## 1. Introduction

The monitoring of soil water content provides essential information that is necessary to mitigate the impact of natural disasters like floods or droughts, and it is also a valuable tool for a precise irrigation, which, in turn, can bring reductions in water consumption [1,2]. There are several methods used to measure soil moisture that can be divided into in-situ methods and remote methods. Among the in-situ methods, electromagnetic techniques have been increasingly attractive, with the time-domain reflectometry (TDR) and frequency-domain reflectometry (FDR)/capacitance techniques being the most common [3]. The application of these methods has the following advantages over other methods used to determine soil moisture: (i) They do not use ionizing radiation and can be used close to the surface of the soil (in contrast to the neutron scattering technique), (ii) they are non-destructive and fast (in contrast to γ ray attenuation and gravimetric techniques), (iii) they allow for the continuous monitoring and recording of soil moisture from dry to near saturated conditions, and (iv) they can be applied to a wide range of soil types [4].

Electromagnetic techniques rely on the measurements of dielectric properties of materials [5]. Relative dielectric permittivity $\varepsilon^{*}$ is a complex number that can be written as:

(1) $$\varepsilon^{*}\left(f\right)=\varepsilon^{\prime }\left(f\right)-j\varepsilon^{\prime \prime }\left(f\right)$$

where the real ($\varepsilon^{\prime }$) and imaginary ($\varepsilon^{\prime \prime }$) parts of dielectric permittivity depend on frequency (f) of the applied electromagnetic wave.

It is recognised that $\varepsilon^{\prime }$ in soil mainly depends on volumetric water content (θ), and, to a lesser degree, on other soil parameters such as mineralogical properties and particle-size distribution. The imaginary part $\varepsilon^{\prime \prime }$ depends on the dielectric loss ($\varepsilon_{d}$) and on the bulk electrical conductivity ($\sigma_{b}$) according to the following formula:

(2) $$\varepsilon^{\prime \prime }=\varepsilon_{d}+\frac{\sigma_{b}}{\omega \varepsilon_{0}},\;\omega =2\pi f$$

where $\varepsilon_{0}\approx 8.85\;\cdot 10^{-12}{\;F\;m}^{-1}$ is vacuum dielectric permittivity.

In the literature, there are a number of theoretical and empirical models describing the relation among the θ, $\sigma_{b}$, and electrical conductivity of soil solution. One of them is the salinity index model developed in [6], where *S*I is defined as:

(3) $$SI=\frac{\partial \sigma_{b}}{\delta \varepsilon_{a}}$$

where $\varepsilon_{a}$ is the apparent dielectric permittivity of soil.

The application of dielectric techniques in order to estimate soil moisture content has resulted in the development of an increasing number of sensors working at various frequencies from kHz up to several GHz [4]. TDR sensors, operating in a frequency band of 1–2 GHz, measure dielectric permittivity with a high accuracy. However, they are still too expensive to be commonly used. The FDR and capacitance methods represent an alternative to the TDR technique because of their lower cost. Nevertheless, these sensors usually operate at a single frequency in the range of 20–300 MHz. At these frequencies, soil complex permittivity can exhibit several polarization phenomena, including bound water, double-layer, and Maxwell–Wagner effects [7,8]. These phenomena depend not only on water content and salinity but also on other properties of soils such as mineralogy and particle-size distribution.

From a user point of view, a dielectric sensor should be able to operate in different types of soil and should be capable of adapting to changing environmental conditions. The manufacturers usually offer commercial sensors with the calibration equation that relates θ to either the dielectric permittivity or the output magnitude unit of the sensor. However, in many cases, a soil-specific calibration is recommended, even though it is challenging for many users to perform. In general, dielectric sensors can be calibrated in two ways. One way, called one-step calibration, is to directly calibrate the sensor against soil water content like in the gravimetric method of soil moisture determination [9]. However, applying a direct calibration is often too labour-intensive and time-consuming. The use of a two-step calibration seems to be a more promising method [10]. The first step of this calibration relies on measurements of reference (calibration) materials with known values of $\varepsilon^{*}$. The second step is the application of the relations between θ and $\varepsilon^{*}$ in the form of an empirical [11] or semi-theoretical model. This approach allows for the determination of θ. However, the results obtained from different dielectric sensors may vary.

The electromagnetic sensors are usually evaluated using the determination of permittivity in the soil over a range of water contents [12]. These studies are useful for the demonstration of the general water content measurement capability of specific soils. The obtained results are often misleading, and the effects arising from bound water or salinity can be disguised by used soil-specific calibration. The response of the sensors is varied among soil type, temperature, and salinity [13]. The reliable comparison of the performance of dielectric sensors requires the use of repeatable and stable reference materials with well-known dielectric properties, reflecting the dielectric spectrum of soil. The “ideal” reference materials should be non-toxic, inexpensive, and easily accessible. Due to the fact that many soils (e.g., especially fine-textured soils) exhibit multiple relaxation phenomena including Maxwell–Wagner effects at low frequencies, the reference materials should also reproduce dielectric relaxation and ionic conductivity behaviour [10]. Several authors have developed sensors calibration techniques by using calibration media such as air and liquids like water, 2-isopropoxyethanol, dioxane, ethylene glycol, methanol, and ethanol [10,14,15,16]. The use of liquids as calibration media has several advantages: (i) no air gaps and density variations, (ii) the ability to calibrate multiple sensors in a wide range of permittivity (from 2 to 35), and (iii) the possibility to separate sensor- and soil-specific effects (air or water gaps, metal electrode polarization, and dielectric dispersion at a specific frequency) [17]. Nevertheless, most of the calibration liquids, such as dioxane and 2-isopropoxyethanol, are harmful to health. Additionally, liquids do not represent all of the loss mechanisms and relaxations that occur in the soil in the range of the minimum to the maximum moisture level. An alternative is the use of grainy solids or powdery materials that reflect the dielectric permittivity spectrum of a natural soil, e.g., minerals (kaolinite, bentonite), glass beads, talc, sand, and calcium carbonate under different moisture conditions [14,18]. However, the knowledge of the dielectric complex permittivity spectrum of these materials under different moisture conditions, including different salinity levels, is required. The application of glass beads and talc as reference materials has several advantages: (i) they regular spherical particles with a known and repeatable diameter, (ii) they have easy access, (iii) they allow for their easy recovery from a mixture, and (iv) a mixture of these materials with water has low hazard potential and there are no effects on the sensor [19]. Additionally, it is worth noting that talc is characterized by low dielectric losses [20].

The aim of the study was to measure the complex dielectric permittivity of talc, glass beads, and their mixtures under different moisture and salinity levels. This paper presents the following:

- Electromagnetic simulations of the seven-rod probe with different dimensions using the Ansys HFSS software (Version 19.2.0, ANSYS, Inc. Southpointe, Canonsburg, PA, USA).
- Measurements of the $\varepsilon^{\prime }$ and $\varepsilon^{\prime \prime }$ values of talc, glass beads, and glass beads with 5% and 10% of talc under different moisture and salinity conditions (electrical conductivities of 0.5, 1.0, and 1.5 S·m^−1^) using the short seven-rod probe connected to a PNA-L N5230C VNA (Agilent Technologies, Santa Clara, CA, USA) in the operational frequency range of the probe.
- Comparison between $\varepsilon^{\prime }$ and θ with well-known calibration equations at a several frequencies.
- Estimation of repeatability of the materials under test.
- Evaluation of the salinity index model.

## 2. Materials and Methods

### 2.1. Seven-Rod Probe

**Figure 1:**
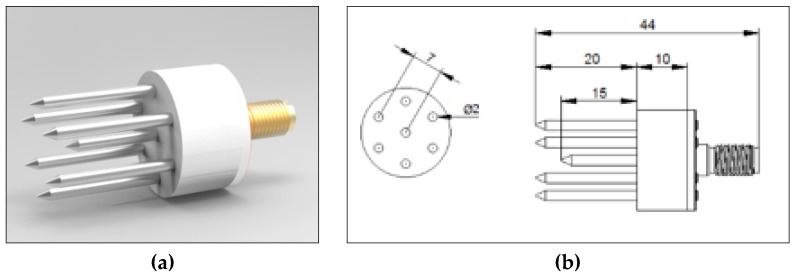
The short seven-rod probe used in this work: (**a**) probe picture and (**b**) respective dimensions of the probe (in mm).

In this study, complex dielectric permittivity spectra were measured using a short seven-rod probe with 15 and 20 mm long inner and outer rods, respectively, with a measurement volume of about 2800 mm^3^. The probe was manufactured at the Laboratory of Dielectric Spectroscopy of the Institute of Agrophysics PAS, Lublin, Poland (Figure 1a,b). The probe used in this work (a short seven-rod probe) was a modified version of the probe described in [21]. The construction of the seven-rod probe enabled us to measure soil moisture in a well-defined volume. Because the operating frequency range of the probe shown in [21] was 200 MHz, in order to extend its operational frequency range, another construction of probes with shorter central and outer rods was proposed. Probes with 25 and 30 mm long central and outer rods and 15 mm and 20 mm long central and outer rods, respectively, were tested by the numerical simulations using the finite element method (FEM) in the Ansys HFSS software [22]. The simulated probes were surrounded by tested material in the form of a cylinder of 70 mm in height and 15 mm in diameter filled with calibration media (air, water, and ethanol) and isopropanol as a verification liquid. More details about the simulation space and the applied model were presented in [21]. The extraction of complex permittivity spectrum from the simulated reflection coefficient ($S_{11}$) was performed using the following bilinear equation:

(4) $$\varepsilon^{*}=\frac{c_{1}S_{11}-c_{2}}{c_{3}-S_{11}}$$

where $c_{1}$,$\;c_{2}$, and $c_{3}$ are calibration constants determined in the open-water-liquid (OWL) calibration [23,24].

The value of the $\varepsilon^{\prime }$ of isopropanol (verification liquid) obtained from Equation (4) was based on the simulation data. Next, the obtained $\varepsilon^{\prime }$ of isopropanol obtained from the two probes with different dimensions were compared with the data shown in [21] for a probe with 40 mm long inner rod and with the data of isopropanol found in the literature [25].

### 2.2. Materials Characteristics and Experiment

**Figure 2:**
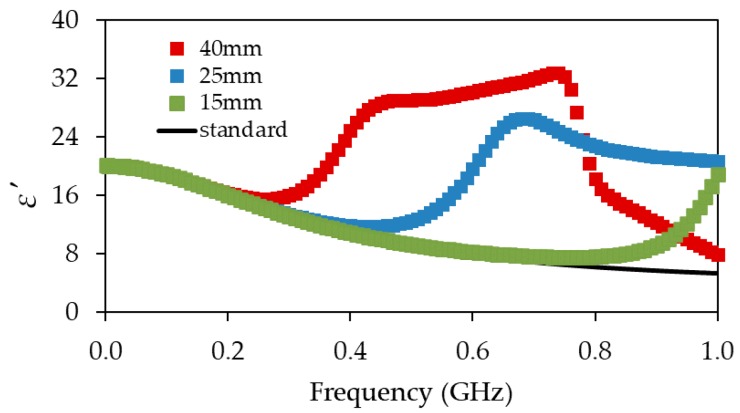
Comparison of $\varepsilon^{\prime }\;$ (real electric permittivity) values for isopropanol obtained from digital simulations for different lengths of the probes: 40 and 45 mm, 25 and 30 mm, and 30 and 20 mm central and outer rods [21], respectively. The black solid line corresponds to the Debye model of isopropanol dielectric spectrum obtained from the literature (T = 20 °C) [25].

The glass beads, 90–106 µm (Fuji Manufacturing Industries, Japan), and mixtures of glass beads with 5% and 10% of talc (Sigma Aldrich) were moistened with distilled water (2 × 10^−4^ S·m^−1^) and with three potassium chloride (KCl) solutions of electrical conductivities of ($\sigma_{KCl})$ 0.5, 1.0, and 1.5 S·m^−1^. The measurements of dielectric properties of the mixture of glass beads with 5% of talc with distilled water were performed in three independent repetitions. Additionally, six samples with different moisture contents were obtained for talc. The measurements were conducted using the short seven-rod probe (Figure 1) connected to a PNA-L N5230C VNA (Agilent Technologies) over the frequency range from 1 to 500 MHz (Figure 2) at 20 °C. At those frequencies, an OWL calibration and the complex dielectric permittivity extraction procedure described above were performed using methanol as the calibration liquid. The weight of both air-dry glass beads and talc did not change after drying at 105 °C for 24 h. This suggested that these materials do not absorb water. A mass of 60 g material (talc, glass beads or glass beads with talc) was mixed with a designated amount (in gram) of liquids in order to obtain 6 different values of moisture contents from 2% to 3% of volumetric water content to near saturation. Next, the sample was transferred to a vial (50 mL) where the volume of each measured sample was about 30,800 mm^3^. After this experiment, all samples were dried in the laboratory oven in 105 °C in order to obtain the dry mass of the samples [26]. The volumetric water content was calculated for all samples. In the next step, the θ*-*$\varepsilon^{\prime }$ calibration curves in the form of linear functions of dielectric permittivity were fitted to the obtained data. Additionally, the salinity index (*SI*) was calculated.

## 3. Results and Discussion

The shortening the rods of the probe with respect to the solution from [21] enabled us to extend the measurement frequency range. The simulation of the $\varepsilon^{\prime }$ values of isopropanol obtained from the short seven-rod probe with the 15 mm central rod agreed with data obtained from the literature [25] up to 500 MHz (Figure 2), which was also confirmed by the low value of root-mean-square error (0.024) between the simulated $\varepsilon^{\prime }$ value and the known reference permittivity of isopropanol. The similar root mean square error (RMSE) (0.025) was obtained up to 200 MHz for the probe with a 40 mm central rod, as presented in [21].

**Figure 3:**
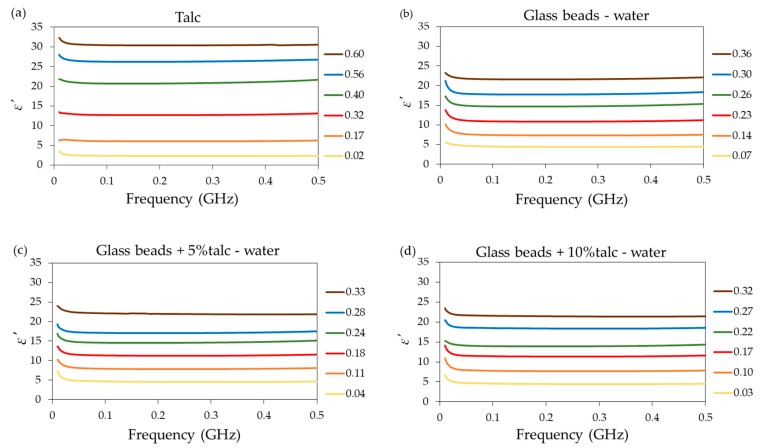

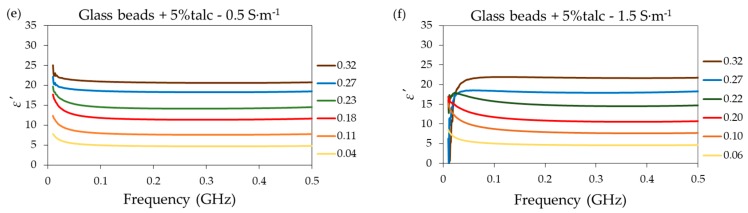
The $\varepsilon^{\prime }$ values of (**a**) talc, (**b**) glass beads, glass beads with (**c**) 5% and (**d**) 10% of talc moisturized with distilled water, and glass beads with 5% of talc moisturized with KCl solutions of (**e**) 0.5 and (**f**) 1.5 S·m^−1^ conductivities over the frequency range from 1 to 500 MHz (selected data). The legends present the θ (volumetric water content) value of samples.

The sample $\varepsilon^{\prime }$ spectra of selected materials obtained using the short seven-rod probe over the frequency range from 1 to 500 MHz are presented in Figure 3.

Despite the fact that the simulation gave the results up to 500 MHz that were consistent with the literature, in the case of the measurements, the upper working frequency of the probe was limited to 400 MHz because a slight increase of $\varepsilon^{\prime }$ was observed above 400 MHz for several samples, which is not a physical behaviour. Additionally, some scattering of the $\varepsilon^{\prime }$ values was observed at low frequencies. This scattering was more visible in the case of samples with higher water and salt contents. As expected, for each sample, the value of $\varepsilon^{\prime }$ increased with an increasing water content [21,27,28]. Among all the samples, the highest value of $\varepsilon^{\prime }$ for the saturation point was obtained for talc. According to the literature data, talc and glass beads exhibit hydrophobic character. This means that water does not adsorb inside the particles of these materials. However, water can be absorbed on the particles’ surface due to the forces cohesion and adhesion [29]. The highest value of $\varepsilon^{\prime }$ for the saturation point of talc can be explained by its smaller particle size as opposed to those of the glass beads.

In the next step, the θ–calibration curves were fitted in the form of a linear function of the square root of dielectric permittivity, as given by the following equation:

(5) $$\theta =A\sqrt{\varepsilon^{\prime }}-B$$

**Figure 4:**
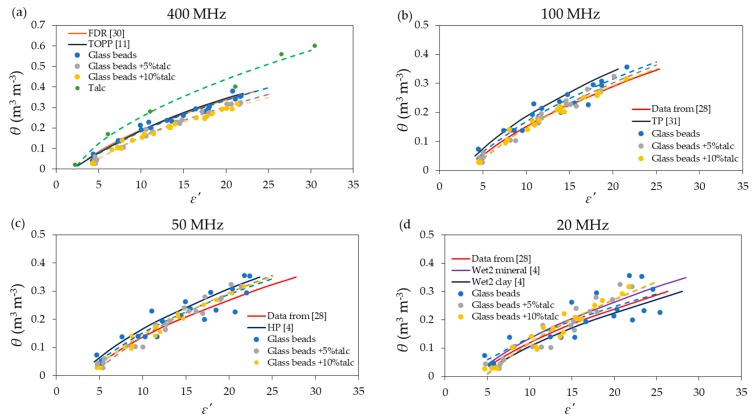
Relations between $\varepsilon^{\prime }$ and θ at (**a**) 400, (**b**) 100, (**c**) 50, (**d**) 20 MHz. Solid lines represent the reference calibration functions given in [4,11,28,30,31], dashed lines represent the fitted functions according to Equation (5), where the $\sqrt{\varepsilon^{\prime }}$ linear functions were presented in $\varepsilon^{\prime }$ coordinates.

The fitting equations were obtained for each of the materials, except for talc, at selected frequencies for data including distilled water and all sodium chloride solutions. For talc, it was only obtained for distilled water. Figure 4 shows the relations between $\varepsilon^{\prime }\;$ values for the materials under test and an appropriate reference equation available in literature:

Wet2 sensor calibrations for mineral and clay soils at 20 MHz [4]:

(6) $$\theta =0.099\sqrt{\varepsilon^{\prime }}-0.178\;\left(\mathrm{mineral}\right),\;\theta =0.091\sqrt{\varepsilon^{\prime }}-0.182\;\left(\mathrm{clay}\right)$$

HydraProbe (HP) calibration for mineral and loam soils at 50 MHz [4]:

(7) $$\theta =0.109\sqrt{\varepsilon^{\prime }}-0.179$$

ML3 ThetaProbe (TP) calibration for mineral soils at 100 MHz [31]:

(8) $$\theta =0.119\sqrt{\varepsilon^{\prime }}-0.190$$

Calibration function for an FDR sensor obtained in the frequency range of 390–480 MHz [30]:

(9) $$\theta =0.113\sqrt{\varepsilon^{\prime }}-0.167$$

Topp’s equation obtained for TDR [11]:

(10) $$\theta =-5.3\cdot 10^{-2}+2.92\cdot 10^{-2}\varepsilon_{a}-5.5\cdot 10^{-2}\varepsilon_{a}^{2}+4.3\cdot 10^{-6}\varepsilon_{a}^{3}$$

Calibration equation for 10 soils at 20, 50, and 100 MHz [28]:

(11) $$\theta =0.92\sqrt{\varepsilon^{\prime }}-0.172\;\left(20\;\mathrm{MHz}\right),\;\theta =0.101\sqrt{\varepsilon^{\prime }}-0.182\;\left(50\;\mathrm{MHz}\right),\;\theta =0.106\sqrt{\varepsilon^{\prime }}-0.184\;\left(100\;\mathrm{MHz}\right)$$

The application of pure talc as a reference material was under consideration. However, as can be seen in Figure 4a, talc stood out from the rest of considered materials and calibration curves. Moreover, it was difficult to remove the short seven rod probe from the talc sample, especially in the case of higher water content. The abandonment of further measurements of talc mixed with KCl solutions was caused by those two reasons. For the same value of θ, the value of $\varepsilon^{\prime }$ for tested materials increased according to $\varepsilon^{\prime }{}_{\mathrm{talc}}$ < $\varepsilon^{\prime }{}_{\mathrm{glass}\;\mathrm{beads}}$ < $\varepsilon^{\prime }{}_{\mathrm{mixture}\;\mathrm{glass}\;\mathrm{beads}\;\mathrm{and}\;\mathrm{talc}}$. The addition of talc to the glass beads caused a diminished air space volume between the glass beads, which, in turn, decreased the water content at saturation for the mixture of glass beads with talc. Additionally, both glass beads and the mixture of glass beads with talc gave results closer to well-known reference calibration functions. In the case of glass beads, the data points were more scattered in comparison to glass beads with the talc addition. Additionally, there were no significant differences between application 5% and 10% of talc. The fitted parameters of Equation (5), including the *R*^2^ and RMSE values obtained for each material under different moisture and conductivity conditions for selected frequencies, are presented in Table 1.

Generally, the materials under test gave high values of *R*^2^, which increased with an increase of frequency. The highest value of *R*^2^ was observed in the glass beads with 10% of talc. It also occurred that the maximum value of the RMSE was obtained at 20 MHz. For each material, the RMSE decreased with an increase in frequency. The smallest differences between the maximum and minimum values of the RMSE among the tested materials was observed for glass beads with 10% of talc.

**Figure 5:**
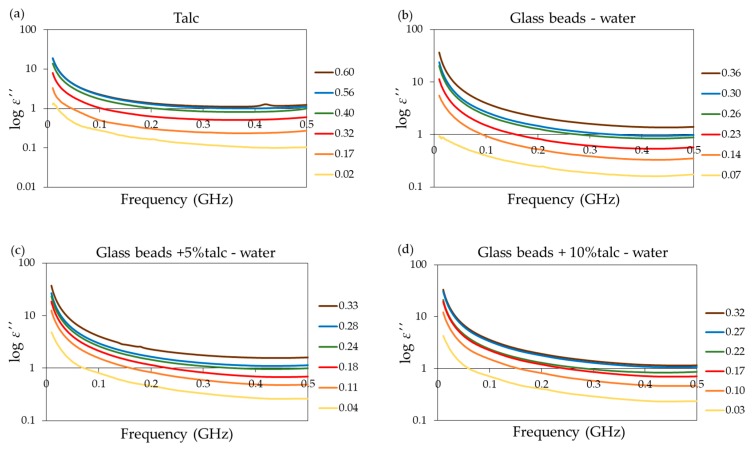

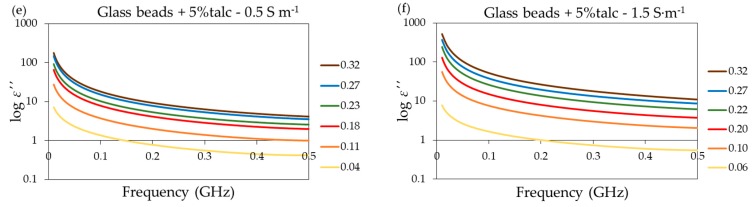
The $\varepsilon^{\prime \prime }$ (imaginary electric permittivity) values of (**a**) talc, (**b**) glass beads, glass beads with (**c**) 5% and (**d**) 10% of talc moisturized with distilled water, and glass beads with 5% of talc moisturized with KCl solutions of (**e**) 0.5 and (**f**) 1.5 S·m^−1^ conductivities over the frequency range from 1 to 500 MHz (selected data). The legends present the θ value of samples.

The $\varepsilon^{\prime \prime }\;$ spectra of selected materials are presented in Figure 5.

**Figure 6:**
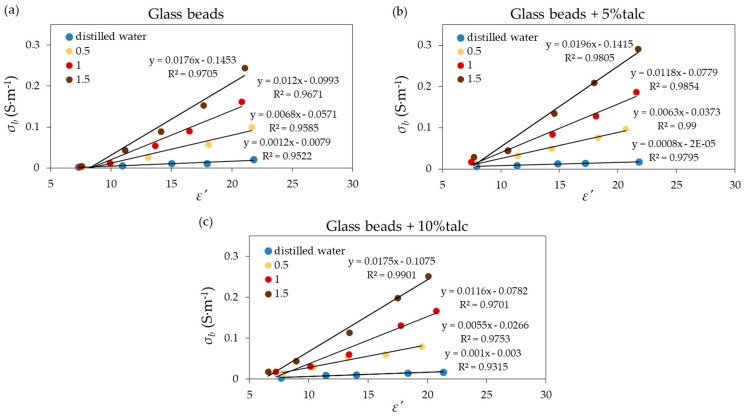
Relation between the bulk electrical conductivity ($\sigma_{b}$) and $\varepsilon^{\prime }$ of the measured (**a**) glass beads, (**b**) glass beads with 5% of talc, and (**c**) glass beads with 10% of talc moistened with distilled water and KCl solutions. The value of $\sigma_{KCl}$ (S·m^−1^) is given in the legend. The linear regression equation and *R*^2^ for each material is also presented.

As expected, the addition of salt increased the value of the $\varepsilon^{\prime \prime }$ samples. Talc exhibited the lowest dielectric loss, which is characteristic of this material. In the next step, $\sigma_{b}$ was obtained using the Levenberg–Marquardt algorithm as implemented in the MATLAB (Version 7.12.0.635, MathWorks Inc., Natick, MA, USA) lsqnonlin function for nonlinear least square fitting. Then *SI* values for measurement materials were calculated using Equation (3). Following [32], $\varepsilon_{a}$ was substituted with $\varepsilon^{\prime }$. For the calculation, the samples with $\varepsilon^{\prime }$ < 3.5 were excluded from analysis, because the salinity index model does not work in the case of dry samples. The relations between $\sigma_{b}$ and $\varepsilon^{\prime }$ at 400 MHz, presented in Figure 6 for the tested materials, were linear for all applied solutions with high value of *R*^2^ > 0.95. The highest *R*^2^ was obtained for glass beads with the addition of talc.

**Figure 7:**
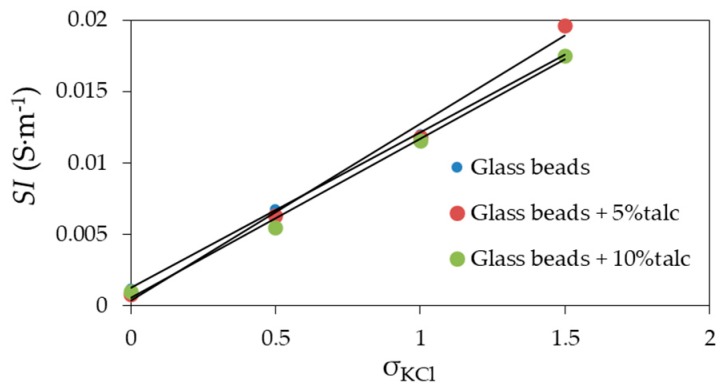
Relation between *SI* (salinity index) and $\sigma_{KCl}$ of the measured glass beads, glass beads with 5% of talc, and glass beads with 10% of talc moistened with distilled water and KCl solutions. The value of $\sigma_{KCl}$ is given in the legend.

Figure 7 shows the relation between *SI* and $\sigma_{KCl}$ for materials under test. The relations were highly linear for each considered material, which was represented by high *R*^2^ (Table 2), where ***l*** is the slope of a relation between *SI* and the $\sigma_{KCl}$. For the glass beads with 5% of talc, ***l*** = 0.0124 ± 0.000194, which matched the *l* = 0.0128 value that was calculated using TDR for sand [6]. Similar results were also obtained for glass beads with 0.26 mm [33].

Next, the repeatability of the dielectric properties of the most promising reference material was examined. In this case, the mixture of glass beads with 5% of talc moistened with distilled water was measured in three independent repetitions. It was calculated that the differences in the value of *ɛ’* between repetitions did not exceed 1%. This showed that the glass beads with talc met repeatability requirements, which is the one of important parameter for a reference dielectric material.

## 4. Conclusions

This paper presented the dielectric properties of glass beads and mixtures of glass beads with talc. It was concluded that the addition of talc to the glass beads provided more repeatability and stability of the material, which was observed through the diminished scattering of the data points in comparison to the results from the glass beads alone. The fitting equations obtained for the mixture of glass beads with talc, especially for the 5% addition of talc, gave results close to well-known reference calibration functions. It was also observed that pure talc stood out from the rest of the tested materials and the calibration curves, which makes pure talc a less optimal calibration material than the aforementioned mixtures. Additionally, the addition of 5% of talc also provided the highest linear correlations between $\sigma_{b}$ and $\varepsilon^{\prime }$, which is an important assumption of the salinity index model. It was observed that the slope *l* of *SI* versus the $\sigma_{KCl}$ relation was similar to the slope obtained for sand. In summary, the obtained data indicated that the glass beads with the addition of 5% of talc can be a promising material for the calibration and verification of dielectric methods and devices for measuring soil moisture. The addition of clay material to a mixture of glass bead with 5% of talc is planned in future studies in order to provide better modelling of low-frequency dielectric relaxation phenomena.

## Figures and Tables

**Table 1:** Fitted parameters of Equation (5), *R*^2^, and root mean square error (RMSE).

Material	*f* (GHz)	A	B	R^2^	RMSE
Talc-water	0.02	0.1442	0.2196	0.9825	0.2311
	0.05	0.1431	0.2068	0.9824	0.2339
	0.10	0.1426	0.2021	0.9823	0.2351
	0.40	0.1405	0.1902	0.9838	0.2288
Glass beads	0.02	0.0841	0.1292	0.7030	0.6504
	0.05	0.1038	0.1759	0.8752	0.3413
	0.10	0.1121	0.1862	0.9390	0.2113
	0.40	0.1163	0.1843	0.9757	0.2172
Glass beads and 5% talc	0.02	0.1176	0.2540	0.8986	0.2585
	0.05	0.1185	0.2368	0.9636	0.1543
	0.10	0.1168	0.2201	0.9776	0.1184
	0.40	0.1129	0.2002	0.9886	0.1166
Glass beads and 10% talc	0.02	0.1169	0.2498	0.9375	0.1967
	0.05	0.1147	0.2264	0.9806	0.1117
	0.10	0.1114	0.2062	0.9869	0.0919
	0.40	0.1076	0.1878	0.9900	0.0883

**Table 2:** Slope *l* of *SI* versus the $\sigma_{KCl}$ relation for glass beads, glass beads with 5% of talc, and glass beads with 10% of talc using linear regression.

Material	*l*	Std. Error	*R* ^2^
Glass beads	0.0109	1.48·10^−4^	0.9998
Glass beads and 5%talc	0.0124	1.93·10^−4^	0.9918
Glass beads and 10%talc	0.0111	1.55·10^−4^	0.9958
